# Multi-scale factors influencing the characteristics of avian communities in urban parks across Beijing during the breeding season

**DOI:** 10.1038/srep29350

**Published:** 2016-07-11

**Authors:** Shilin Xie, Fei Lu, Lei Cao, Weiqi Zhou, Zhiyun Ouyang

**Affiliations:** 1 University of Science and Technology of China(USTC), Faculty of Life Sciences, Hefei 230026, China; 2 Chinese Academy of Sciences(CAS), State Key Laboratory of Urban and Regional Ecology, Research Center for Eco-Environmental Science, Beijing 100085, China

## Abstract

Understanding the factors that influence the characteristics of avian communities using urban parks at both the patch and landscape level is important to focus management effort towards enhancing bird diversity. Here, we investigated this issue during the breeding season across urban parks in Beijing, China, using high-resolution satellite imagery. Fifty-two bird species were recorded across 29 parks. Analysis of residence type of birds showed that passengers were the most prevalent (37%), indicating that Beijing is a major node in the East Asian–Australasian Flyway. Park size was crucial for total species abundance, but foliage height diversity was the most important factor influencing avian species diversity. Thus, optimizing the configuration of vertical vegetation structure in certain park areas is critical for supporting avian communities in urban parks. Human visitation also showed negative impact on species diversity. At the landscape level, the percentage of artificial surface and largest patch index of woodland in the buffer region significantly affected total species richness, with insectivores and granivores being more sensitive to the landscape pattern of the buffer region. In conclusion, urban birds in Beijing are influenced by various multi-scale factors; however, these effects vary with different feeding types.

Human populations in urban environments are estimated to reach 5 billion by 2025, representing 65% of the world’s total population^1^. Therefore, it is essential to understand and minimize the impact of the process of urbanization on the fragmentation and islanding of urban bird habitats^2^,^3^,^4^,^5^, which are associated with less vegetation cover and more artificial surfaces^6^,^7^. Urbanization impacts urban avian communities by causing a decline in bird species richness and diversity, in parallel with an increase in overall bird density^8^. Consequently, as the level of urbanization increases, the similarity of bird species composition increases^9^.

Many researchers are dedicated to investigating the mechanisms of these changes, and have identified a variety of influencing factors at different spatial scales. Local-scale factors are considered to play a more decisive role than regional factors in bird species richness^10^, particularly for species with restricted distributions that require specific habitats^11^. For example, high vegetation cover and more complex vegetation structure have a significantly positive impact on avian communities^12^,^13^,^14^. Mature vegetation provides a natural barrier between birds and pedestrians, which reduces the negative impact of human disturbance on urban birds, assisting their ability to adapt to urban environments^15^. Forest composition and structure may represent the most crucial factors influencing avian species abundance and diversity^16^. However, patch area is generally assumed to be the most important factor^17^,^18^, because larger parks often exhibit richer landscape types, lower edge effects, and, therefore, more bird species^19^. Furthermore, human presence is also generally considered to have a negative impact on avian species abundance and diversity^18^,^20^.

Several landscape-level influencing factors also affect avian communities in parks. For example, the urban matrix around forest patches reduces the fitness of park forests for birds^18^,^21^. This result contrasts with naturally heterogeneous regions, where the landscape surrounding the patch contributes little towards explaining relative bird abundance^22^. Landscape fragmentation exhibits significant effects on avian community structure^23^, including the number of roads and quantity and density of buildings, with different bird species exhibiting different levels of sensitivity to such fragmentation^24^,^25^. Studies in Hong Kong found that the landscape contagion index, patch density, and landscape evenness index in the buffer zone of urban parks significantly affected avian community structure; however, different bird guilds with different feeding types (termed autoecology) were found to respond differently to landscape factors^26^,^27^.

Urban parks represent important green spaces in built-up areas, and are major hotspots of biodiversity. Urban green spaces may affect avian communities at multi-spatial scales, which contain complex spatial patterns^28^. Yet, few studies have examined the factors that affect avian communities at different scales due to a lack of historical data and restricted technical conditions^29^,^30^. For instance, most studies to date have used low-resolution satellite imagery within buffer zones, with the risk of generating relatively large errors in the results of landscape classification^27^.

Beijing is the capital of the People’s Republic of China, and is a highly developed city. There are rich fragmented patches in the urban landscape of Beijing, with each patch containing a high population density and major human activity. At present, research on the factors influencing avian communities in Beijing is limited to the patch level, including the positive impacts of park area, plant species richness, and the structural configuration and the negative impacts of human disturbance on avian communities^31^,^32^,^33^. Most of these studies focused on the differences among different seasons, and may fail to fully reflect avian community characteristics during the breeding season, along with important influencing factors. Beijing also has issues with major smog in winter, with smog only being absent during strong winds, which interfere with bird surveys. In fact, even at the national scale, research remains limited on the response of birds to urbanization in China^8^,^34^. About one-third of all bird species found in China have been detected in Beijing^35^, with this city also falling along an important migratory flyway in East Asia.

Therefore, it is important to study factors that influence avian communities in Beijing at both the patch- and landscape-level to enhance the management of green areas in this city and other similar cities. Thus, this study focused on assessing: (1) the avian community characteristics of urban parks in Beijing during the breeding season; (2) factors influencing avian communities in urban parks in Beijing during the breeding season at the patch level (park area[TA], foliage height diversity [FHD], and humans visitations [HV]) and landscape level (artificial surface-to-woodland ratio, landscape contagion index, largest patch index of woodland, landscape diversity index, and landscape evenness index); (3) whether park area or FHD are the most important factor influencing species abundance and diversity; and (4) whether there are significant differences in the responses of different feeding types to multi-scale influencing factors.

## Results

### Avian Community Characteristics

**Figure 1:**
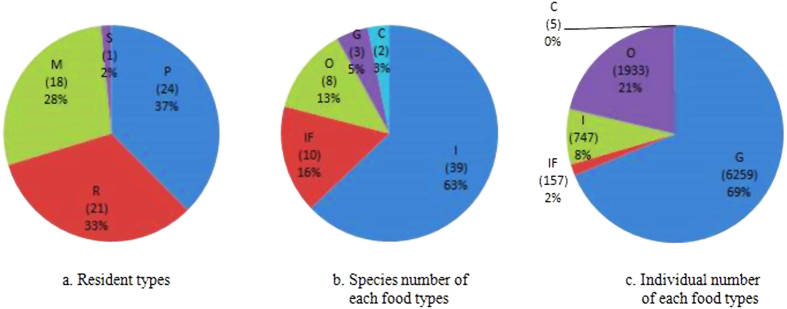
Seasonal status and food types of birds recorded during field surveys. Number of species and individuals from each group is shown in parentheses following the abbreviated name. Species were categorized by residency status and food types. For status: R = resident, M = migratory, P = passenger, S = straggler. For food type: G = granivore, I = insectivore, O = omnivore, IF = insectivore–frugivore, C = carnivore.

**Figure 2:**
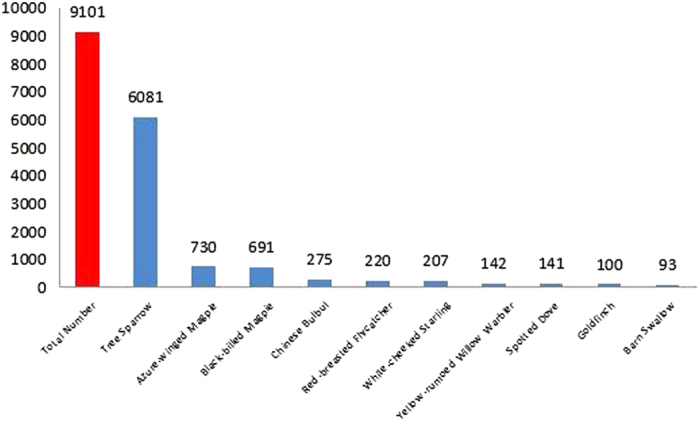
The 10 most abundant bird species recorded during field survey.

In total, we detected 52 species and 9101 individuals in the field survey, which belonged to nine orders and 23 families, accounting for 14.6% of the total number of bird species recorded (355 species) in Beijing^35^ (Table S1). Resident, passenger, and migratory species each accounted for approximately one third of the total species recorded. Stragglers accounted for the lowest percentage, with only the Tree Pipit *Anthus trivialis* being recorded (Fig. 1). Like species number, the greatest number of individuals was represented by Passeriformes (8685 birds, 95.43% of the total number). Within Passeriformes, the Tree Sparrow had the largest number of individuals (accounting for 67.12% of the total number). Figure 2 shows the 10 most abundant bird species. During this survey, we found two species of grade II national protected birds: Sparrow Hawk *Accipiter nisus* (order: Accipitriformes) and European Hobby *Falco subbuteo* (order: Falconiformes).

### Bird Species Richness

The values of the avian community index and patch-level habitat factors are presented in Supplementary Table 2. Spearman correlation analysis results (Table 1) showed that total bird species richness was significantly affected by TA (r = 0.874, p < 0.01), 5URB% (r = −0.403, p < 0.05) and 5WLPI (r = 0.383, p < 0.05). A model was built using the multiple linear regression of bird species richness versus PC1 (environment variable 1 from PCA results), PC2, TA, FHD, and HV. The model indicated that TA (standardized B = 0.890) was the only key factor for bird species richness. The model produced good regression results (correlation coefficient: R = 0.890; R^2^ = 0.793; adjusted R^2^ = 0.772; and ANOVA analysis showed that the model explain about 79.27% of the total variance, while F = 38.227, sig < 0.01). The regression equation of the model (with the standardized coefficients) was expressed as follows:


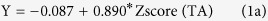


**Figure 3:**
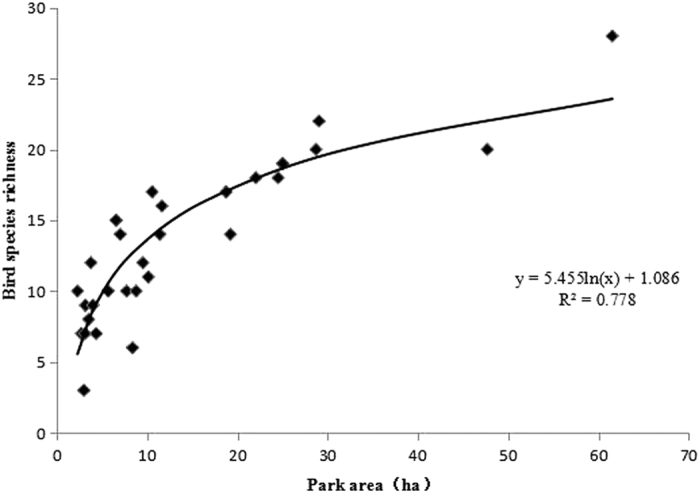
Total area—bird species richness correlation curve.

The species-area curve supports the important role of park area for bird species abundance (Fig. 3).

### Bird Biodiversity

The Spearman correlation analysis results (Table 1; Supplementary Table 2) showed that avian community diversity was significantly affected by TA (r = 0.492, p < 0.01) and FHD (r = 0.454, p < 0.05). Two models were built to examine the effects of PC1, PC2, TA, FHD, and HV on bird diversity using multiple linear regression. We excluded model 1 because its R^2^ < 0.5. Model 2 indicated that FHD (standardized B = 1.005) and HV(standardized B = −0.588) were the most important factor for avian diversity, while other factors were excluded, including TA. The regression results of the model were relatively good (correlation coefficient: R = 0.807; R^2^ = 0.652; adjusted R^2^ = 0.575; and ANOVA analysis showed that the model explain about 65.20% of the total variance, while F = 8.431, sig < 0.01). The regression equation of the model (with the standardized coefficients) was expressed as follows:


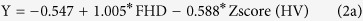


### Species Evenness

Spearman correlation analysis showed that bird species evenness presented significant positive correlation with FHD (r = 0.456, p < 0.05; Supplementary Table 2). No linear regression relationship was detected between multiple independent and dependent variables, making it unsuitable for constructing a linear regression model.

### Avian Community Characteristics of Different Feeding Guilds and Influencing Factors

The avian community characteristics of the different feeding guilds are shown in Supplementary Table 2. We detected 39 insectivores in the bird surveys, accounting for 75% of the total number of bird species. In addition, we detected 10 insectivore–frugivores (19.23%), eight omnivores (15.38%), three granivores (5.77%), and two carnivores (3.85%). The percentage of the different feeding guilds was ordered: granivores (6259, 68.77%) >omnivores (1933, 21.24%) >insectivores (745, 8.19%) >insectivore–frugivores (157, 1.73%) >carnivores (5, 0.0005%). Figure 2 shows the species and numerical composition characteristics of each feeding guilds.

The factors that influenced the different feeding guilds were evaluated using Spearman correlation analysis (Table 1) and multiple linear regression (stepwise regression) (Table 2). Insectivore–frugivores and carnivores were excluded from these analyses as they were only detected in a few parks. Correlation analysis with respect to feeding guild showed that TA significantly impacted the species diversity (r = 0.860, p < 0.01; r = 0.416, p < 0.05) and evenness (r = 0.455, p < 0.05; r = −0.416, p < 0.05) of insectivores and granivores, along with the species richness (r = 0.662, p < 0.01) of granivores. Also, species diversity of insectivores significantly infected by HV (r = –0.454, p < 0.05), 5URB% (r = –0.439, p < 0.05) and 5WLPI (r = 0.398, p < 0.05).

The regression models showed that TA has a major influence on the species richness of granivores (standardized B = 0.522) and the species diversity of insectivores (standardized B = 0.847). Environmental variable PC1 (standardized B = −0.777 or −0.625), which represent the contagion index and SHEI within the buffer region, was also found to have an important effect on the species richness of granivores.

## Discussion

This study showed that the plant composition of urban parks is of greater importance than park area for species diversity. This information is important, because even small parks could potentially support high bird biodiversity, which would also enhance the recreational experience of human visitors, towards which urban parks are primarily tailored.

Interestingly the number of bird species recorded in this survey only accounted for 14.6% of the total number of bird species recorded in Beijing^35^. This low value may be explained because we excluded mountainous birds from the study, because we focused on the urban center of Beijing. In addition, this study focused on breeding season. Therefore, almost all wintering birds were excluded from our records. Resident, passenger, and migratory birds each accounted for approximately similar proportions of individuals (about one third each), indicating that the structure of avian community was relatively even with respect to the composition of residential types in Beijing’s urban parks. However, among the three residential types, passengers accounted for the highest percentage (37%), supporting that Beijing is a major geographic node in the East Asia–Australasian Flyway.

We found that insectivores were the most common feeding guild (39 species, 75%). Thus, the number of insectivores determined the total number of bird species to a large extent in the current study. The number of insectivores also reflected the number and species diversity of insects, which are the major food of insectivores. Thus, insects are a key factor regulating the distribution pattern of insectivores, which is important when protecting birds or attempting to improve bird diversity through targeted management actions^36^. Only three bird species were recorded throughout all of our 29 parks: Tree Sparrow *Passer montanus*, Magpie *Pica pica*, and Light-vented Bulbul *Pycnonotus sinensis*. This result may be due to the fact that most bird species have specific life-history requirements^37^, with only a few urban parks meeting these specifications. Many birds fly to many parks for food, but only breed at specific sites^38^, such as the two grade II national protected species recorded in this study (these species were observed hovering over several parks, but only bred in Xiaoyue Country Park).

At the patch level, both the species–area correlation curve, Spearman correlation analysis and multiple linear regression analysis results showed that the influencing factor TA was positively correlated with avian species richness. Specifically, as park area increased, the number of bird species increased. This finding supports the general conclusions of previous studies^11^,^18^,^39^. However, the model regarding total avian species diversity showed that FHD played the most decisive role on species Shannon diversity index. This result differs to that obtained by previous studies, which suggested that park area is the most important factor^40^,^41^. Our results may differ to these previous studies because parks with high FHD means more kinds and quantity of plant foods, thus could support relatively more evenly distributed avian communities of different feeding types. Also, lower FHD value means more serious visual disturbance, which could also result to the decline of bird species diversity^42^. This phenomenon could also be explained by the fact that the FHD in Beijing’s urban parks was relatively low as a whole when compared with other multi-scale factors. In other words, FHD limits the maximum extent of the avian community. The key role of FHD shows that the vegetation structure must be optimal, particularly the vertical structure, to improve bird species diversity in urban parks. Therefore, in the future planning of urban green spaces in urban regions where the cost of land is high, managers should place greater focus on the configuration of vegetation structure in limited green space areas to enhance the biodiversity and protection of bird species. Also, HV had negative impact on bird species diversity, which consist with the results of previous studies^43^,^44^.

TA had different levels of impacts on species richness and diversity of different feeding guilds, with insectivores and granivores being the most impacted groups. Thus, an increase in TA would be more beneficial to insectivores and granivores compared with other feeding guilds. The possible mechanism underlying this relationship is that the smaller the park area, the greater effect of edge habitats^45^, disrupting foraging by insectivores. Furthermore, insectivores are generally small, timid passerine birds, with small parks representing sub-optimal habitat. In fact, there was a significant negative correlation between the species richness of insectivores and human visits in this study. Zhou *et al*.^20^ found that granivores are positively affected by noise and negatively by foliage height diversity in the wintering season. Our results may have differed because we only detected three urban adapted granivore species; namely, Tree Sparrow, Spotted Dove *Streptopelia chinensis*, and Oriental Turtle Dove *Streptopelia orientalis*. The food sources of these three species are partially provided by human-beings (e.g., rice, maize, and leftovers). Studies have shown that bird feeding behavior is correlated with the financial status of residents^46^. The upscale residential districts are situated near to large urban parks in Beijing; thus, residents living around large urban parks are more likely to provide food for birds. Consequently, TA had a positive impact on the distribution of granivores. In addition, our results showed that TA had a negative impact on the species evenness of granivores, possibly because larger parks are more likely to support rare avian species, which are few in individual number, thus result to the decreasing of species evenness.

At the landscape level, avian community characteristics are significantly correlated with certain landscape factors. Zhou *et al*.^27^ found that habitat evenness and largest patch index for woodland at the 400-m scale and contagion index at the 400- and 1000-m scales have a strong influence on the distribution pattern of birds. In this study, we found that 5URB% and 5WLPI, significantly affected bird species richness. This result indicates that the urban region was highly urbanized, and that urban parks were isolated into green islands by urban buildings and roads, resulting in individual parks failing to meet all the demands of the life-history of urban birds. Yet, the green belt between urban parks (e.g., roadside trees, roadside grass, and even isolated trees) now represents an important green corridor in the urban landscape, alleviating the pressure on birds in urban parks due to severe human disturbance, to some extent. Therefore, we recommend that future urban planning and management place a stronger focus on landscape configuration, especially habitat connectivity^41^ in the buffer zones of urban parks, in addition to the parks themselves, to protect birds.

Overall, the results of our analyses showed that insectivores and granivores are more sensitive to landscape pattern of buffer region. As a major geographic node of the East Asian–Australasian Flyway, Beijing is of great value in the conservation of birds, particularly migratory species and insectivores. Therefore, we recommended that managers retain as much large-scale natural vegetation as possible in future urban planning, along with increasing the number of urban green areas, including parks, and improving the configuration of vertical vegetation structure in existing parks. Also, we suggested that park managers restrict human visitation among peak periods to reduce the negative effects of human disturbance on avian species diversity. All of these actions would contribute towards conserving existing bird biodiversity in areas gradually being encompassed by urban space. In conclusion, this study presents novel information about how different factors influence bird biodiversity at different scales, providing a basis on which to optimize the planning of future green spaces to protect bird biodiversity in built-up areas.

## Methods

### Study Area

Beijing is located in the northernmost part of North China Plain (39°38′–41°05′ N, 115°24′–117°30′ E). The average altitude of the city is 43.5 m above sea level. The history of the city of Beijing extends back more than three thousand years, while its history as a capital city extends back more than 850 years. The region exhibits a typical northern temperate semi-humid continental monsoon climate, with hot and rainy summers, cold and dry winters, and a short spring and fall. The urban area of Beijing has a characteristic concentric loop structure, and the gradient of urbanization gradually declines as loop number increases (from 2 to 6). The resident population reached 21.705 million by the end of 2015, with population density gradually decreasing with increasing loop number^47^. The green belt area within the 5^th^ ring (urban region) accounts for 32.8% of the total urban area^48^. Urban parks throughout the city are the most important green spaces in Beijing. These parks are managed by full-time staff. However, the management of these parks is mainly oriented towards human use, rather than species protection^49^,^50^. Therefore, research is needed to improve the functioning of urban parks in Beijing to maintain bird diversity.

**Figure 4:**
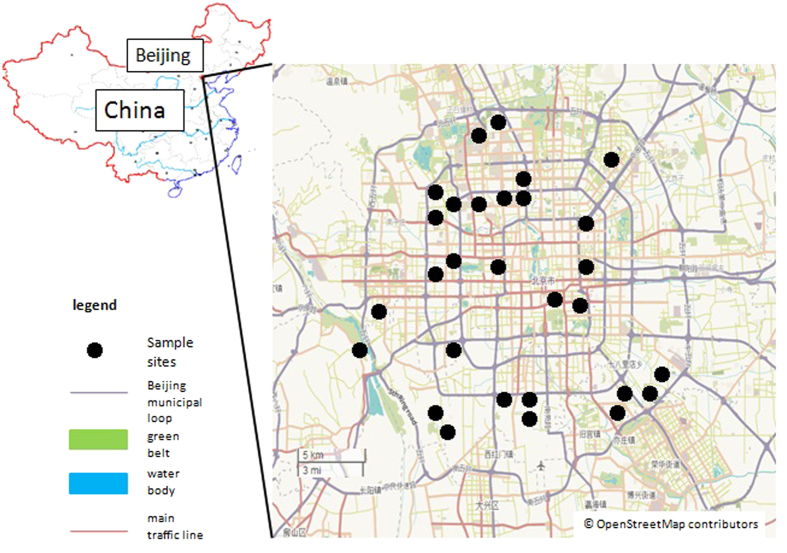
Locations of the 29 selected parks in the urban region of Beijing, China. All parks are located within the 5^th^ loop (urban region), except for Xiaoyue Country Park. The Beijing municipal loop starts from the center (with an initial number of 2), moving outwards (the outermost number is 6). The base map is OpenStreetMap © OpenStreetMap contributors (URL: http://www.openstreetmap.org/export#m ap = 12/39.8602/116.3507&layers = H), The cartography in the OpenStreetMap map tiles is licensed under CC-BY-SA 2.0 (www.openstreetmap.org/copyright). And the map was processed by ArcGIS 10.2(URL: http://www.esri.com/).

Twenty-nine parks ranging in size from 2.27 to 61.53 ha were selected within the fifth loop of Beijing (except for Xiaoyue Country Park, which is located at the outer margin of the fifth loop). All surveyed parks were separated by the urban matrix. Significant gradients in area and location (indicated by the loop number) (Fig. 4) existed among the surveyed parks.

### Bird and Human Visitor Census

During the breeding season (May 1, 2015 to July 23, 2015), birds were surveyed using the fixed line transect method^51^ once or twice per month in each park. Surveys were performed only at time intervals when birds are active (06:30 to 10:30 in the morning and 16:00 to 18:00 in the afternoon), under fine weather conditions, and at a wind speed <30 km/h^27^. The investigator advanced along the line transect at a speed of 1–2 km/h and recorded the birds that were visually observed or heard within 25 m to the front and on both sides of the line transect^16^. Birds that crossed the line transect above treetop height and those that hovered high in the sky were not recorded. During the bird survey, the number of human visitors within the sampling region was recorded. To reduce statistical error between different investigators, the bird survey was only performed by a single investigator (First author).

### Vertical Vegetation Structure Survey

Intercept points were chosen at 100-m intervals along the bird survey route in each sampled park. At each point, a transverse line perpendicular to the bird survey route was chosen. The line was 50 m long (25 m on each side of the bird survey route). Thus, the range of the vegetation structure survey coincided with that of the bird survey. Then, observation points were chosen at 5-m intervals along each transverse line. The presence of leaves at different vertical height levels (0–1, 1–2, 2–5, 5–10, 10–20, and 20–30 m) was observed and recorded at each point, using a 5-m-long pole as visual reference^14^. To avoid subjective estimation errors, the survey of the vertical vegetation structure was also completed by a single investigator (First author)^26^. Finally, FHD was obtained using the Shannon diversity index calculation^52^.

### Landscape Analysis

Landscape analysis was performed using high-resolution IKONOS remote sensing imagery (bands 1, 2, 3, and 4; resolution, 1 m). Images were obtained in September 2012. First, a complete IMG image within the fifth loop of Beijing was acquired via band fusion and splicing using ERDSA IMAGINE 2010. Next, IMG images of the sampled parks were obtained by cropping along the boundary line using the crop function of ENVI. IMG images of the 500-m and 1000-m radius buffer zones outside the park boundaries^53^,^54^,^55^ were obtained using the buffer function of ArcGIS 10.2. The images were then subjected to sophisticated classification using the high-resolution image classification software eCognition 8.7.1 (five types in total, including woodland, grassland, wetland, water bodies, and artificial surfaces). Because wetlands were only distinguished in buffer zones of Dashiqiao Park and the CCTV Tower Park, we did not analyze this landscape type any further in our study. During the classification process, we compared the original image with Google Earth and Google Street View maps. After completing the classification, we combined field surveys to test the obtained accuracy (>89%). ASC files were exported after qualification. As the landscape analysis software fragstats 3.3^56^ only identifies GRID files, we used ArcGIS 9.0 to convert the ASC files into GRID files before the final landscape analysis. Target indices of the landscape analysis are shown in Table 3.

### Data analysis

Bird species richness B was assumed to be equal to n, with n being the total number of bird species recorded in the survey of each surveyed park. The bird biodiversity index H′ was calculated using the Shannon-Wiener index formula:


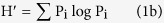


Individual density was calculated as:


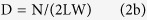


where N is the number of birds recorded in the belt transect, L is the length of the line transect (m), and W is the width of the line transect (m).

The number of visitors to the park was calculated as:


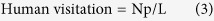


where Np is the average monthly number of human visitors.

All birds were divided by residential type into resident, passenger, migratory, and straggler species. With the exception of one wader (*Tringa ochropus*) recorded in Hongbo Park, all birds were classified by feeding habits as insectivores (I), granivores (G), insectivore–frugivores (IF), omnivores (O), and carnivores (C)^57^. Then, species richness and the Shannon diversity index were calculated for each feeding guild.

All data were subjected to a normality test before statistical analysis. Because many landscape factors (independent variables) showed high autocorrelation and the number of landscape factors was relatively large (12), establishing a model using direct regression would lose practical significance. Therefore, we first conducted a principal component analysis (PCA) of 12 landscape-level independent variables and selected the first two principal components (PC1 and PC2) with an eigenvalue of greater than 1 as environmental variables. According to the absolute value of the score coefficients obtained through PCA, we interpreted PC1 as the landscape contagion index (10CTAG) and landscape evenness index (10EVEN) in the 1000-m radius buffer zone; PC2 was interpreted as the proportion of woodland (5/10WOD%) and the highest patch index of woodland (5/10WLPI) in the 500-m and 1000-m radius buffer zones. Environmental variables PC1 and PC2 are shown in Table 4.

Next, we conducted Spearman correlation analysis with original multi-scale variables, while landscape level factors were represented by PC1 and PC2, combining with park area (TA), FHD, and HV to do regression analyses with various avian community indices (bird species richness, diversity, and evenness). Before multiple linear regression, the standardization (Zscore) of variables were conducted, and stepwise regression was used as the regression method. Then, correlation and regression analyses were performed with respect to bird guild feeding types.

All statistics were completed using SPSS 22.0 Statistics, including Principal Component Analysis, Spearman correlation analyses and regression analyses.

## Additional Information

**How to cite this article**: Xie, S. *et al*. Multi-scale factors influencing the characteristics of avian communities in urban parks across Beijing during the breeding season. *Sci. Rep.* **6**, 29350; doi: 10.1038/srep29350 (2016).

## Supplementary Material

Supplementary Dataset 1

Supplementary Dataset 2

## Figures and Tables

**Table 1:** Spearman Correlation Analysis results for the multi-scale factors and bird community indexes, only the correlation coefficients of significant influences were displayed, others were represented by “—”, while “*” means significant level (p < 0.05), “**” means very significant level (p < 0.01).

Muiti-scale factor	Species richness	Species diversity	Species evenness	Insectivores			Granivores			Omnivores		
				B	H′	J	B	H′	J	B	H'	J
TA	0.874^**^	0.492^**^	—	—	0.860^**^	0.455^*^	0.662^**^	0.416^*^	−0.416^*^	—	—	—
FHD	—	0.454^*^	0.456^*^	—	—	—	—	—	—	—	—	—
HV	—	—	—	—	−0.454^*^	—	—	—	—	—	—	—
5URB%	−0.403^*^	—	—	—	−0.439^*^	—	—	—	—	—	—	—
5WLPI	0.383^*^	—	—	—	0.398^*^	—	—	—	—	—	—	—

**Table 2:** Regression analysis results for the multi-scale factors and bird community indexes of insectivores (I), granivores (G), and omnivores (O), “*” means significant level (p < 0.05), “**” means very significant level (p < 0.01).

	R^2^	R^2^_a_	Sig	Regression models
B(I)	0.351	0.287	0.042	
B(G)	0.604 0.861	0.564 0.830	0.003^**^ 0.000^**^	Y_1_ = −0.380–0.777* Zscore (PC1) Y_2_ = −0.437–0.652* Zscore (PC1) + 0.522 Zscore (TA)
B(O)	0.405	0.346	0.026^*^	
H′(I)	0.717	0.689	0.001^**^	Y = −0.063 + 0.847 * Zscore(TA)
H′(G)	—	—	—	
H′(O)	—	—	—	
D(I)	—	—	—	
D(G)	—	—	—	
D(O)	—	—	—	

**Table 3:** Meanings and ranges of landscape indexes in this research.

Landscape indexes	Description	Mean (range)
5URB%10URB%	% of urban constructed land in a 500 and 1000-m radius	65.7(46.0–79.6), 66.3(51.1–78.1)
5WOD%10WOD%	% of woodland in a 500 and 1000-m radius	28.9(17.8–46.6), 27.9(18.4–41.0)
5CTAG/10CTAG	Contagion index in a 500 and 1000-m radius	64.4(53.7–72.9), 64.9(57.0–73.9)
5WLPI/10WLPI	Largest patch index for woodland in a 500 and 1000-m radius	6.7(1.1–25.4), 4.2(0.9–12.4)
5SHDI/10SHDI	Landscape diversity index in a 500 and 1000-m radius	0.8(0.5–1.0), 0.8(0.6–1.0)
5SHEI/10SHEI	Landscape evenness index in a 500 and 1000-m radius	0.6(0.4–0.8), 0.6(0.4–0.8)

**Table 4:** PCA results of landscape indexes.

	First axis (PC1)	Second axis (PC2)
Eigenvalue	7.75	2.41
Relative percent variance (%)	64.61	20.04
Cumulative percent variance (%)	64.61	84.65
Principal component structure for the first two principal components		
5WOD%	−0.11	0.28
10WOD%	−0.05	0.22
5URB%	−0.01	−0.17
10URB%	−0.07	−0.11
5CONTAG	−0.19	0.08
10CONTAG	−0.21	0.10
5WLPI	−0.09	0.24
10WLPI	−0.08	0.24
5SHDI	0.15	−0.01
10SHDI	0.16	−0.02
5SHEI	0.18	−0.07
10SHEI	0.20	−0.07
